# Depletion of CD40 on CD11c^+^ cells worsens the metabolic syndrome and ameliorates hepatic inflammation during NASH

**DOI:** 10.1038/s41598-019-50976-6

**Published:** 2019-10-11

**Authors:** Suzanne Aarts, Myrthe Reiche, Myrthe den Toom, Marion Gijbels, Linda Beckers, Norbert Gerdes, Esther Lutgens

**Affiliations:** 1 Department of Medical Biochemistry, Subdivision of Experimental Vascular Biology, Amsterdam University Medical Centers, location AMC, Amsterdam Cardiovascular Sciences (ACS), University of Amsterdam, Amsterdam, The Netherlands; 2 Department of Pathology and Department of Molecular Genetics, CARIM, University of Maastricht, Maastricht, The Netherlands; 3 Division of Cardiology, Pulmonology, and Vascular Medicine, Medical Faculty, University Hospital Düsseldorf, Düsseldorf, Germany; 4 Institute for Cardiovascular Prevention (IPEK), Ludwig Maximilian’s University (LMU), Munich, Germany

**Keywords:** Obesity, Inflammatory diseases, Immunological disorders

## Abstract

The co-stimulatory CD40-CD40L dyad plays a central role in fine-tuning immune reactions, including obesity-induced inflammation. Genetic ablation of CD40L reduced adipose tissue inflammation, while absence of CD40 resulted in aggravated metabolic dysfunction in mice. During obesity, CD40 expressing CD11c^+^ dendritic cells (DC) and macrophages accumulate in adipose tissue and liver. We investigated the role of CD40^+^CD11c^+^ cells in the metabolic syndrome and nonalcoholic steatohepatitis (NASH). DC-CD40-ko mice (CD40^fl/fl^CD11c^cre^) mice were subjected to obesity or NASH. Obesity and insulin resistance were induced by feeding mice a 54% high fat diet (HFD). NASH was induced by feeding mice a diet containing 40% fat, 20% fructose and 2% cholesterol. CD40^fl/fl^CD11c^cre^ mice fed a HFD displayed increased weight gain, increased adipocyte size, and worsened insulin resistance. Moreover, CD40^fl/fl^CD11c^cre^ mice had higher plasma and hepatic cholesterol levels and developed profound liver steatosis. Overall, regulatory T cell numbers were decreased in these mice. In NASH, absence of CD40 on CD11c^+^ cells slightly decreased liver inflammation but did not affect liver lipid accumulation. Our experiments suggest that CD40 expressing CD11c^+^ cells can act as a double-edged sword: CD40 expressing CD11c^+^ cells contribute to liver inflammation during NASH but are protective against the metabolic syndrome via induction of regulatory T cells.

## Introduction

Obesity is a major risk factor for a variety of diseases including cardiovascular diseases, diabetes, nonalcoholic fatty liver disease (NAFLD) and nonalcoholic steatohepatitis (NASH)^1^. Obesity is defined as the abnormal accumulation of body fat to the extent that it presents a negative effect on health. These days, 13% of the adult population and 7% of the children worldwide are obese^1^. Obesity results in a state of systemic, low-grade inflammation caused by immune cell infiltration and activation in adipose tissue and liver^2,3^. Activation of macrophages, dendritic cells (DCs), natural killer cells, T cells, B cells and adipocytes in the adipose tissue results in cytokine and adipokine release which can lead to obesity-associated metabolic dysregulation, characterized by insulin resistance and dyslipidemia^4,5^.

CD40 is a co-stimulatory molecule that upon binding to CD40L regulates inflammation by activating or suppressing immune cells^6,7^. Deficiency of CD40L in diet-induced obesity ameliorates inflammation of the adipose tissue and reduces insulin resistance and liver steatosis^8,9^. In contrast, genetic deficiency of CD40 in mice fed a high fat diet resulted in worsened insulin resistance and increased adipose tissue inflammation compared to wild-type mice^10–13^. An explanation for the different metabolic phenotypes in the CD40L and CD40 deficient mice can be sought in the cell type specific functions of this co-stimulatory molecule. CD40 is expressed by a variety of cell types that play a role in obesity and metabolic dysfunction including monocytes, macrophages, DCs, B cells, T cells, endothelial cells^14^ and adipocytes^15^.

The integrin CD11c is a DC surface marker, but is also present on B, T and NK cells and subsets of monocytes and macrophages^16^. Increased numbers of CD11c^+^ cells are found in the liver of mice under obese conditions^17^, and liver inflammation contributes to the progression from NAFLD (defined as the presence of ≥5% of hepatic steatosis) to NASH^18^. In humans, adipose tissue CD11c expression is discriminative for crown-like-structure macrophages and the presence of CD11c^+^ macrophages correlates with markers of insulin resistance^19^. CD40 expression was detected on CD11c^+^ adipose tissue cells in obese mice while lowly expressed in lean adipose tissue^20^.

We here investigate the contribution of CD40 on CD11c^+^ cells in the regulation of diet induced obesity and NASH using CD40^fl/fl^CD11c^cre^ mice. We found that CD40 expressing CD11c^+^ cells contribute to diet-induced-obesity (DIO) and NASH in opposing ways. In diet induced obesity CD40 expressing CD11c^+^ cells play a crucial role in protection against obesity-induced ectopic lipid storage and metabolic dysfunction, most likely via induction of regulatory T cells. During NASH, CD40 on CD11c^+^ cells contributes to liver inflammation.

## Results

### *CD40*^*fl/fl*^*CD11c*^*cre*^ mice have increased body weight gain and excess lipid deposition compared to WT mice after high fat diet feeding

**Figure 1:**
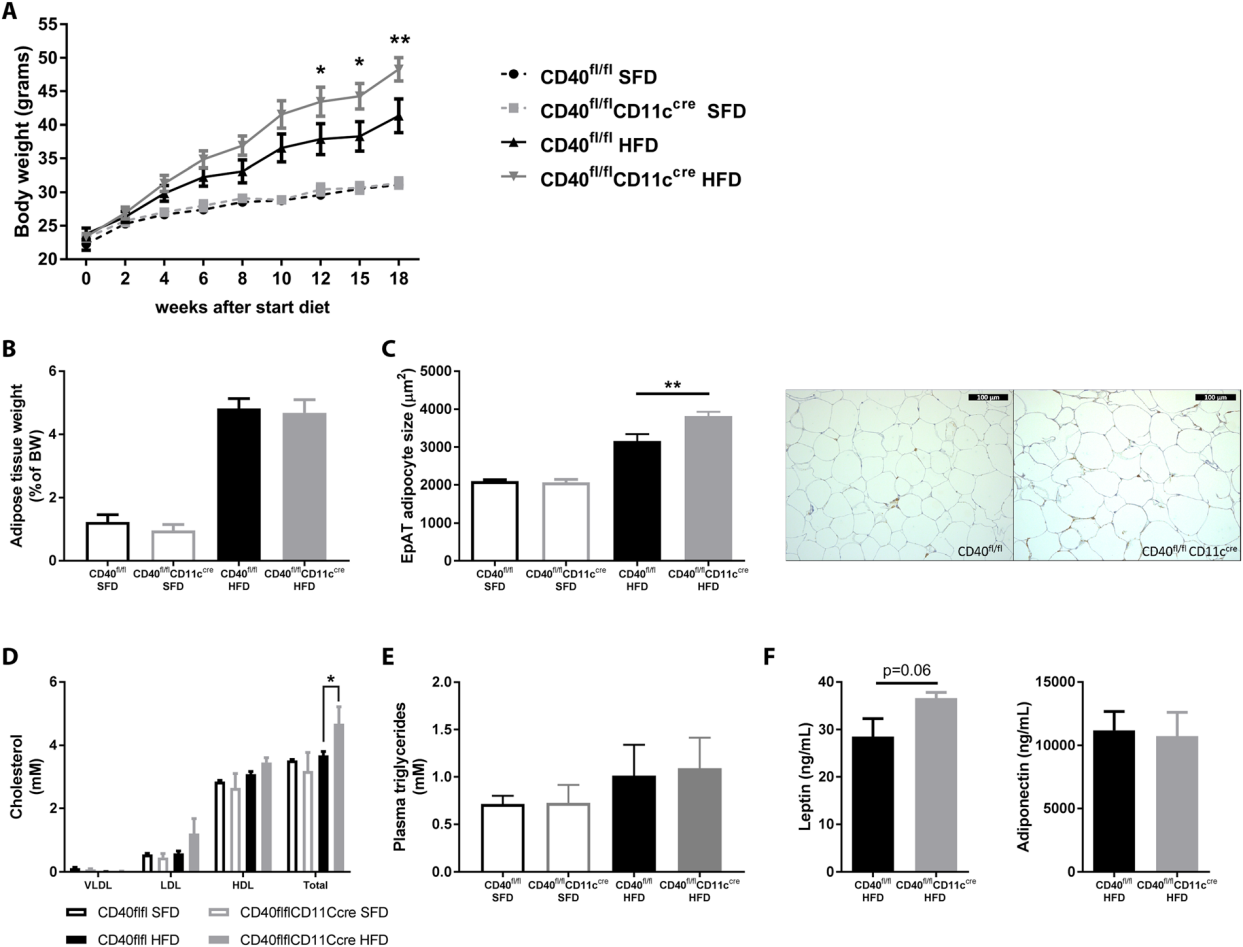
CD40^fl/fl^CD11c^cre^ mice on HFD are more obese than WT mice. (**A**) Body weight gain during 18 weeks of standard (SFD) or high fat diet (HFD) feeding. (**B**) epidydimal adipose tissue (EpAT) tissue weight after 18 weeks of diet. (**C**) EpAT adipocyte size after 18 weeks of diet and representative HE pictures of EpAT of CD40^fl/fl^CD11c^cre^ mice and WT mice fed an HFD (scale bar is 100 µm). (**D**) Total plasma cholesterol and lipid fractions. (**E**) Plasma triglycerides. (**F**) Plasma leptin and adiponectin levels. Data is represented as mean ± SEM. *P < 0.05; **P < 0.01 for comparison between WT and CD40^fl/fl^CD11c^cre^ mice fed the same diet. n = 7/group for SFD, and n = 8/group for HFD.

During the course of DIO, CD40^fl/fl^CD11c^cre^ mice gained more weight than their wild type littermates (two-way ANOVA, p = 0.0012 at week 18, Fig. 1A). This difference was specific for HFD fed mice and was not observed in the mice fed SFD (Fig. 1A). EpAT weight did not differ between CD40^fl/fl^CD11c^cre^ and WT mice (Fig. 1B). However, adipocyte size of HFD CD40^fl/fl^CD11c^cre^ mice was increased by 20.9% compared to WT mice (unpaired t-test, p = 0.0082, Fig. 1C) indicating excess lipid deposition. In line with these results, total cholesterol levels in plasma were significantly higher in CD40^fl/fl^CD11c^cre^ mice compared to WT mice on HFD (two-way ANOVA, p = 0.0476, Fig. 1D). Plasma VLDL, HDL and LDL levels did not differ significantly, but the increase in HDL and LDL levels explain the increase in total cholesterol in the HFD CD40^fl/fl^CD11c^cre^ mice (Fig. 1D). Plasma triglyceride levels did not differ (Fig. 1E). Leptin concentrations in the plasma slightly increased in the CD40^fl/fl^CD11c^cre^ group compared to the WT group on the HFD (unpaired t-test, p = 0.0601, Fig. 1F). Adiponectin levels did not differ between the groups (Fig. 1F).

### CD40^fl/fl^CD11c^cre^ mice display slightly aggravated insulin resistance

**Figure 2:**
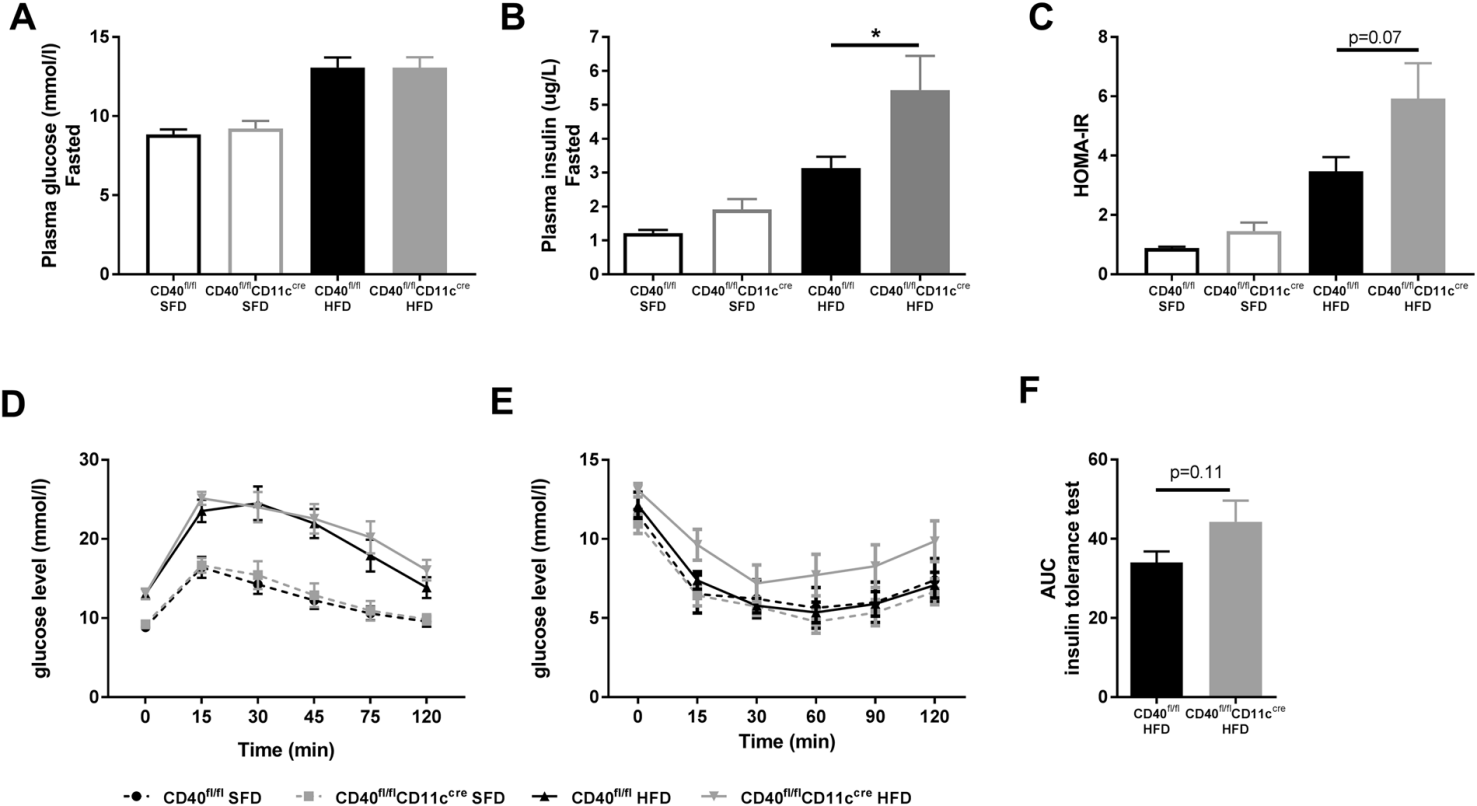
CD40^fl/fl^CD11c^cre^ mice display slightly aggravated insulin resistance. (**A**) Plasma glucose levels after 6 hours of fasting. (**B**) Plasma insulin levels after 6 hours of fasting. (**C**) HOMA-IR. (**D**) Plasma glucose levels measured during the glucose tolerance test (GTT). (**E**) Plasma glucose levels measured during the insulin tolerance test (ITT). (**F**) Area under the curve (AUC) insulin tolerance test. Data is represented as mean ± SEM. *P < 0.05 for comparison between WT and CD40^fl/fl^CD11c^cre^ mice fed the same diet. n = 7/group for SFD, and n = 8/group for HFD.

Saturation of adipocytes can lead to excess lipid storage in other metabolic organs such as liver and muscle^21^, which can result in insulin resistance and abnormal production of insulin^4,22^. Indeed, HFD feeding increased fasted levels of glucose compared to levels of SFD mice, but no differences between the CD40^fl/fl^CD11c^cre^ and WT mice were observed (Fig. 2A). However, fasted plasma insulin levels of HFD CD40^fl/fl^CD11c^cre^ mice had increased 1.7-fold compared to WT mice (unpaired t-test, p = 0.0476, Fig. 2B), indicating compensation of dysfunctional insulin signaling and worsened insulin resistance in CD40^fl/fl^CD11c^cre^ mice. The homeostatic model assessment for insulin resistance (HOMA-IR) shows a tendency towards an increased score in the HFD CD40^fl/fl^CD11c^cre^ mice, implying that CD40^fl/fl^CD11c^cre^ mice display aggravated insulin resistance when fed HFD (unpaired t-test, p = 0.0764, Fig. 2C). Glucose sensitivity was impaired after HFD feeding, but there were no differences between CD40^fl/fl^CD11c^cre^ and WT mice when fed a similar diet (Fig. 2D). Consistent with the increase in fasting insulin levels, a trend towards worsened insulin resistance as measured via an ITT was observed in the CD40^fl/fl^CD11c^cre^ mice fed HFD compared to WT mice (area under the curve of 34.0 (mean) ± 7.9 (SD) in HFD WT mice vs 44.3 ± 15.2 in HFD CD40^fl/fl^CD11c^cre^ mice, unpaired t-test, p = 0.1113, Fig. 2E,F). These data suggest that the excess (ectopic) lipid accumulation in CD40^fl/fl^CD11c^cre^ mice results in slightly worsened insulin resistance in this model.

### Adipose tissue inflammation is not affected in CD40^fl/fl^CD11c^cre^ mice

FACS analysis of the adipose tissue revealed that leukocyte infiltration of the AT in CD40^fl/fl^CD11c^cre^ mice did not significantly differ from WT mice when fed a similar diet (Supplemental Fig. 1A). Histological stainings of the EpAT with CD68 for macrophages and CD3 for T cells confirm these findings (Supplemental Fig. 1B). Further analysis of myeloid and lymphoid subsets also showed no significant differences (Supplemental Fig. 1C,D). This was confirmed by the mRNA transcription profile of inflammatory mediators in EpAT (Supplemental Fig. 2A,B), revealing that absence of CD40 on CD11c^+^ cells does not affect AT inflammation.

### Loss of CD11c^+^CD40 causes the development of liver steatosis during diet-induced obesity

**Figure 3:**
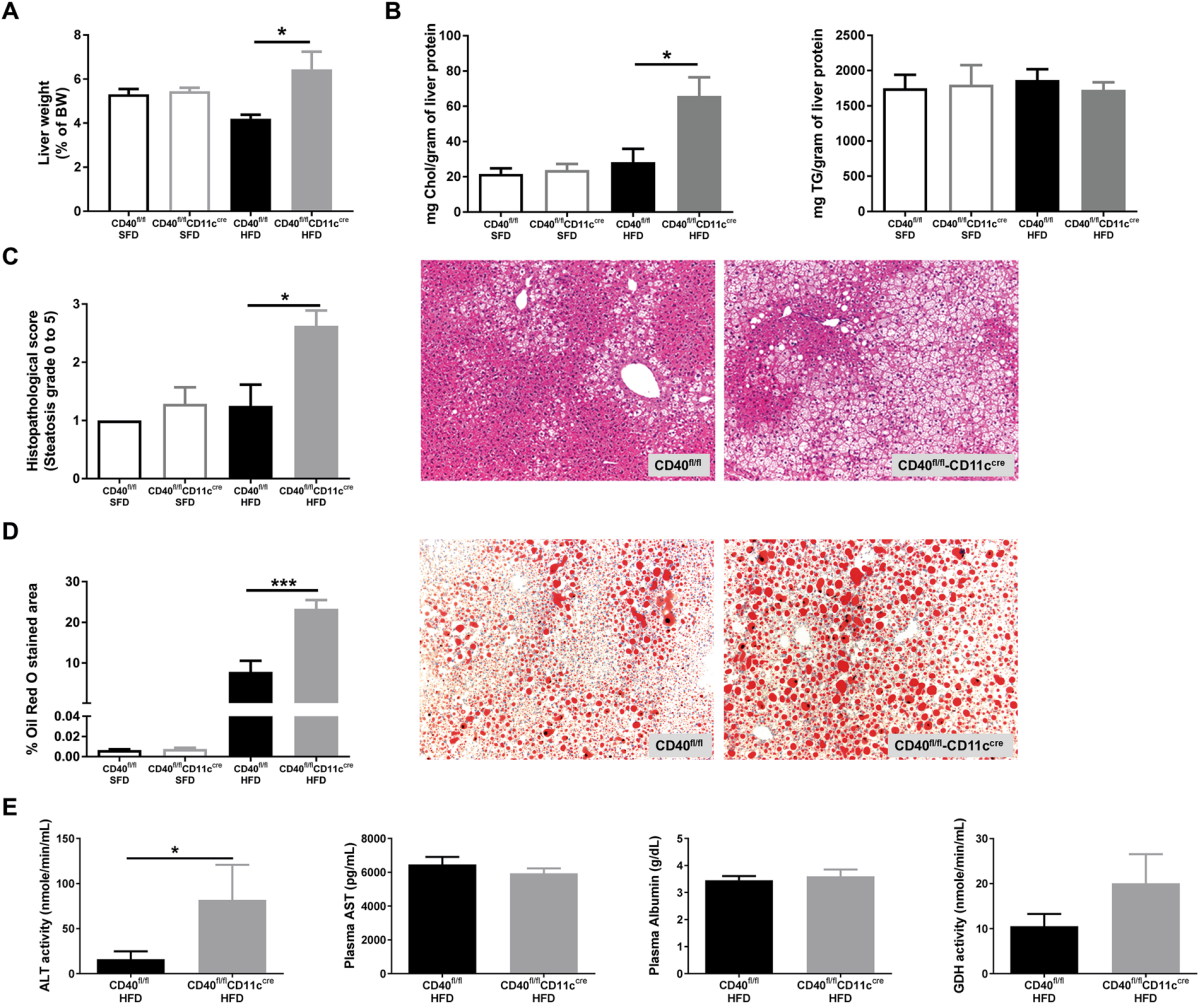
Increased liver steatosis characterizes CD40^fl/fl^CD11c^cre^ mice after 18 weeks of HFD. (**A**) Liver weight (grams). (**B**) Total cholesterol/gram protein and TG/gram protein of liver. (**C**) Histopathological scoring of liver steatosis and representative H&E staining of liver. (**D**) Quantification of liver Oil-red-O (ORO) staining and representative pictures. (**E**) Plasma liver enzymes: alanine aminotransferase (ALT) activity, aspartate aminotransferase (AST) levels, albumin levels and glutamate dehydrogenase (GDH) activity. Data is represented as mean ± SEM. *P < 0.05, **P < 0.01, ***p < 0.001 for comparison between WT and CD40^fl/fl^CD11c^cre^ mice fed the same diet. n = 7/group for SFD, and n = 8/group for HFD.

As decreased storage capacity of the adipose tissue leads to ectopic lipid deposition, we next assessed the severity of obesity-induced hepatic steatosis. In CD40^fl/fl^CD11c^cre^ mice, the percentage liver weight compared to body weight was significantly increased (6.4 ± 2.3% of BW) compared to WT mice (4.2 ± 0.5% of BW) (unpaired t-test, p = 0.0162, Fig. 3A). Liver total cholesterol levels were highly increased in CD40^fl/fl^CD11c^cre^ mice compared to WT mice on the HFD (unpaired t-test, p = 0.0118, Fig. 3B) while triglyceride content was not affected (Fig. 3B). As expected from the high liver cholesterol levels, the grade of steatosis was significantly higher in CD40^fl/fl^CD11c^cre^ mice on a HFD compared to WT mice (median steatosis score of grade 3 in CD40^fl/fl^CD11c^cre^ mice compared to grade 1 in WT mice, Mann-Whitney test, p = 0.0177, Fig. 3C). The increased steatosis was confirmed by ORO staining showing significantly increased lipid accumulation in the liver (Fig. 3D). Quantification of the ORO staining indicated that 7.8 ± 7.7% of the liver of WT mice is steatotic vs 23.3 ± 6.1% of the liver of CD40^fl/fl^CD11c^cre^ mice (mean ± SD), unpaired t-test, p = 0.0005, Fig. 3D). Excess free cholesterol in the liver can accumulate in the hepatocytes, Kupffer cells, and hepatic stellate cells where it can induce cellular toxicity or proinflammatory and profibrotic effects^23^. In order to examine whether these mice suffered from liver damage due to excessive storage of fat in the liver, we measured the presence of liver enzymes and damage related proteins in the plasma that could indicate hepatocellular injury^24^. Significantly increased levels of plasma ALT were found in CD40^fl/fl^CD11c^cre^ mice (Mann-Whitney test, p = 0.0207, Fig. 3E), while AST and albumin levels did not differ from levels found in WT mice on HFD (Fig. 3E). Liver diseases that result from hepatocyte necrosis, and not from inflammation, result in elevated serum GDH levels^25^. GDH activity in CD40^fl/fl^CD11c^cre^ mice was higher compared to the WT mice, although not significant (unpaired t-test, p = 0.1967, Fig. 3E). Sirius Red staining of the liver shows that there is no fibrosis present after 18 weeks of HFD (data not shown), but the increased levels of ALT and GDH in the plasma of CD40^fl/fl^CD11c^cre^ mice indicate that the liver steatosis had caused damage to the hepatocytes of these mice.

### Regulatory T cells numbers are decreased in the liver of CD40^fl/fl^CD11c^cre^ mice with DIO

**Figure 4:**
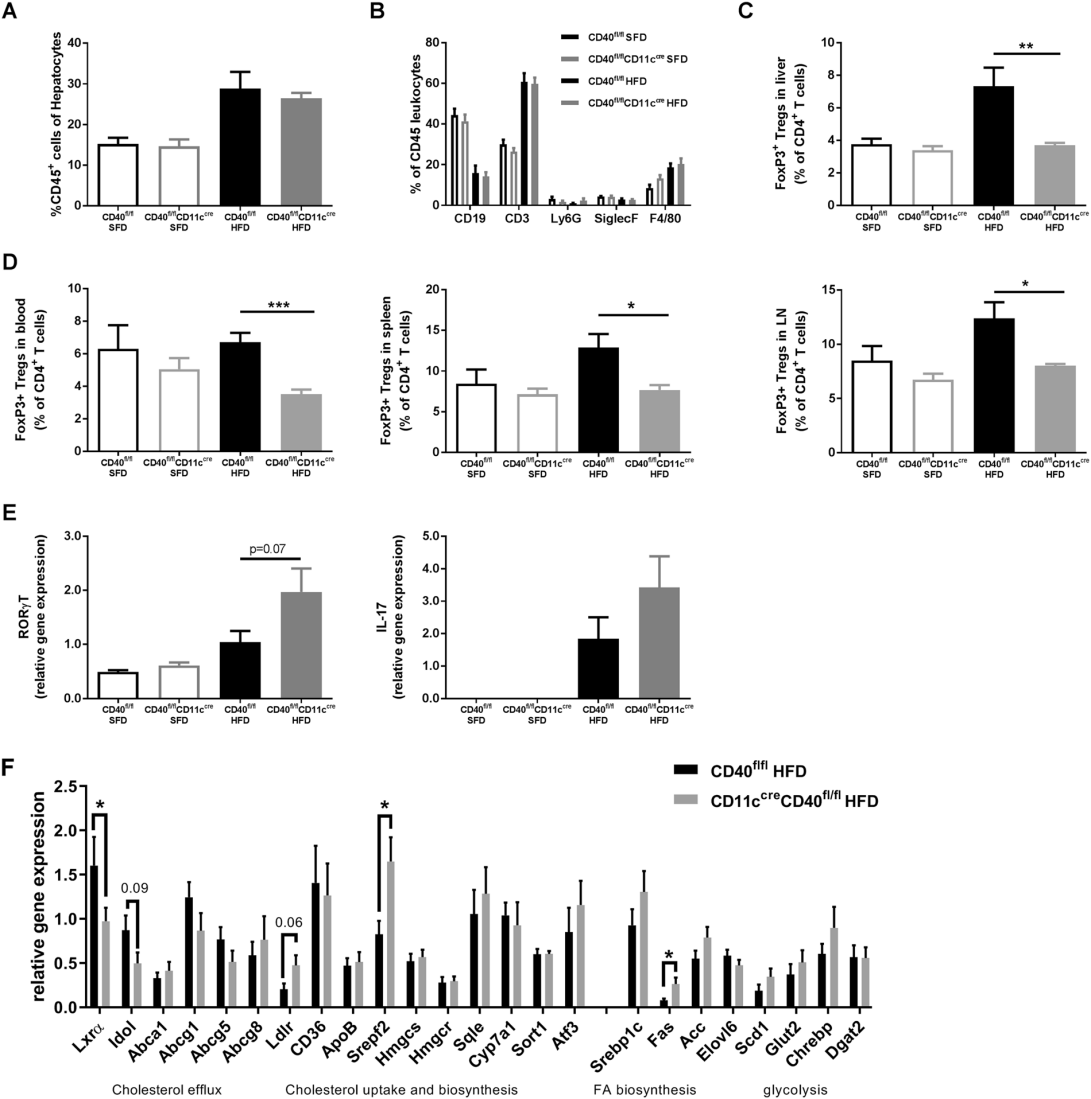
Reduced numbers of regulatory T cells (Tregs) were found in CD40^fl/fl^CD11c^cre^ mice after 18 weeks of HFD. (**A**) Histological quantification of CD45 staining of liver. (**B**) Flow cytometric analysis of leukocyte subsets in the liver. (**C**) % of CD4^+^FoxP3^+^ Tregs in liver. (**D**) Systemic % of CD4^+^FoxP3^+^ Tregs measured in blood, spleen, and lymph nodes (LN). (**E**) liver mRNA expression of RORγT and IL-17. (**F**) mRNA expression of cholesterol metabolism genes in the liver of HFD fed CD40^fl/fl^CD11c^cre^ and WT mice. Data is represented as mean ± SEM. *P < 0.05; **P < 0.01, ***p < 0.001 for comparison between WT and CD40^fl/fl^CD11c^cre^ mice fed the same diet. n = 7/group for SFD, and n = 8/group for HFD.

Liver inflammation was investigated by histopathological quantification of the CD45 staining and showed no differences in number of leukocytes present between the groups that received a similar diet (Fig. 4A). Liver leukocyte subsets were further analyzed by flow cytometry (Fig. 4B,C). The percentage of CD3^+^ T cells in the liver was increased in both the HFD groups compared to SFD mice (Fig. 4B). Interestingly, the percentage of CD4^+^FoxP3^+^ regulatory T cells (Tregs) was significantly lower in the CD40^fl/fl^CD11c^cre^ mice on a HFD compared to WT mice on the same diet (unpaired t-test, p = 0.0067, Fig. 4C). This decrease in Tregs was also observed in blood, spleen and LN of CD40^fl/fl^CD11c^cre^ mice (Fig. 4D). This difference in Treg population was only observed after HFD feeding and was not found in the SFD mice (Fig. 4D). Livers of CD40^fl/fl^CD11c^cre^ mice on a HFD showed a slightly increased expression of RORγt (unpaired t-test, p = 0.0725, Fig. 4E) and IL17 compared to WT mice mRNA (Fig. 4E), indicating increased presence of Th17 cells in the liver of HFD fed CD40^fl/fl^CD11c^cre^ mice.

### Liver cholesterol metabolism gene expression analysis

Absence of CD40 on CD11c^+^ cells caused increased cholesterol levels in liver and blood in obesity. Gene expression analysis of liver mRNA show decreased expression of genes involved in cholesterol secretion (LXRα (unpaired t-test, p = 0.0123) and Idol (unpaired t-test, p = 0.0923, Fig. 4F)), and increased expression in genes related to cholesterol uptake and biosynthesis (LDLr (unpaired t-test, p = 0.0600), and SREBP2 (unpaired t-test, p = 0.0195), Fig. 4F) in HFD fed CD40^fl/fl^CD11c^cre^ mice compared to WT mice suggesting and imbalance in liver cholesterol metabolism in these mice.

### The degree of hepatosteatosis is not different in *CD40*^*fl/fl*^*CD11c*^*cre*^ mice compared to WT mice on a NASH inducing diet

**Figure 5:**
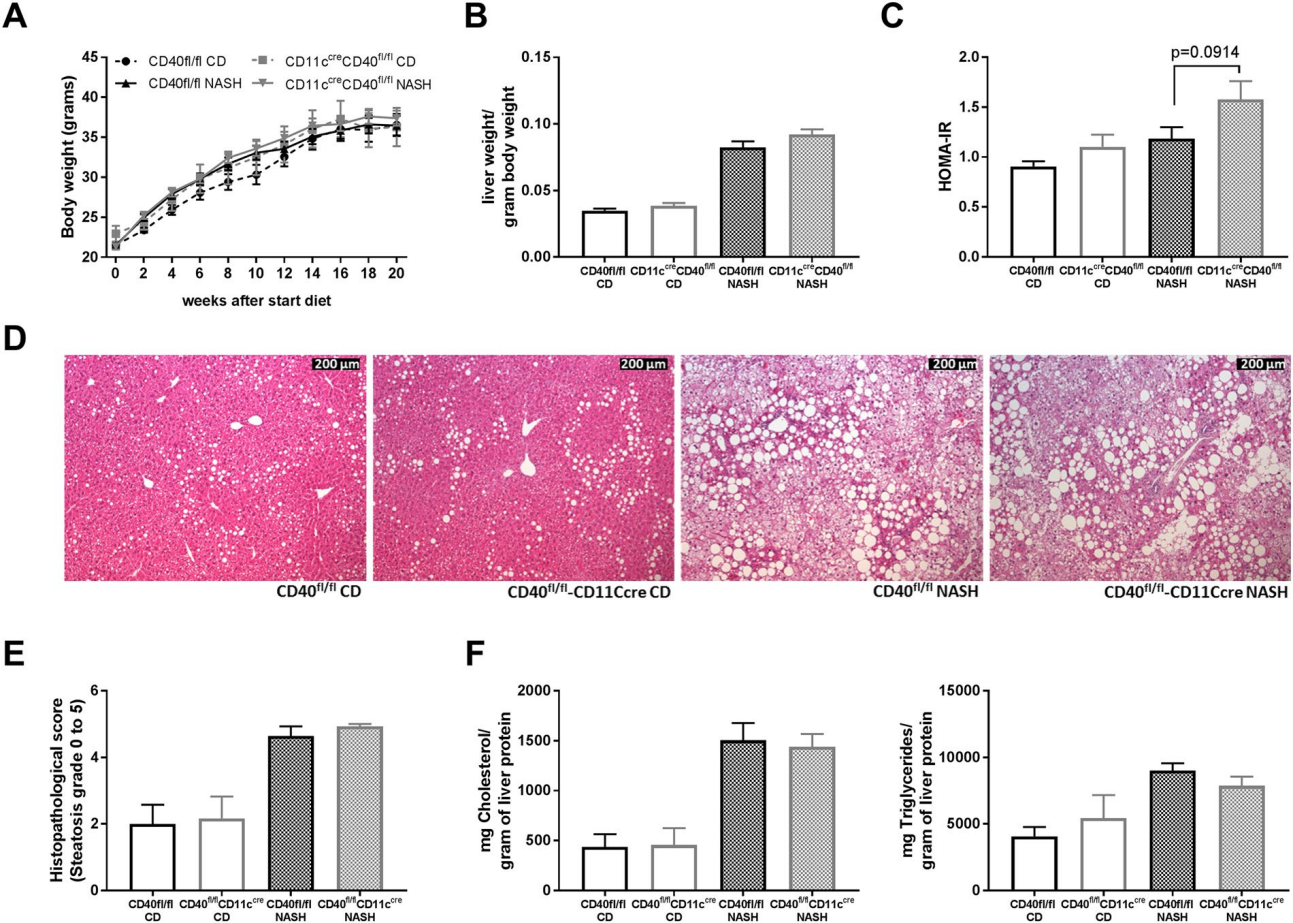
Effect of NASH and CD diet feeding in CD40^fl/fl^CD11c^cre^ and WT mice. (**A**) Body weight. (**B**) Liver weight per gram body weight. (**C**) HOMA-IR. (**D**) Representative pictures H&E staining liver (scale bar is 200 um). (**E**) Mean histopathological steatosis degree. (**F**) Cholesterol and Triglyceride content liver. Data is represented as mean ± SEM. n = 6/group for control diet (CD), and n = 15/group for NASH diet.

As the degree of hepatic inflammation in DIO is rather low, we decided to further investigate the (anti)inflammatory potential of CD11c^+^CD40^+^ cells in a more inflammatory model of hepatosteatosis, and we induced NASH in both genotypes. The NASH inducing diet did not affect body weight compared to the control diet and there were no differences between CD40^fl/fl^CD11c^cre^ mice and WT mice that were fed a similar diet (Fig. 5A). Increased liver weights were observed after feeding the NASH diet, but there was no difference between CD40^fl/fl^CD11c^cre^ mice and WT mice (Fig. 5B). HOMA-IR was slightly increased in CD40^fl/fl^CD11c^cre^ mice fed a NASH diet (score 1.18 ± 0.42 in WT vs 1.58 ± 0.71 in *CD40*^*fl/fl*^*CD11c*^*cre*^ mice, unpaired t-test, p = 0.0914, Fig. 5C). Furthermore, the degree of steatosis was similar in CD40^fl/fl^CD11c^cre^ mice and WT mice on the NASH diet (Fig. 5D,E, representative HE pictures). Liver Cholesterol and TG levels were similar in the different groups on the same diet (Fig. 5F). Fasted plasma glucose measured after 20 weeks of diet was not different between the groups (Supplemental Fig. 3A). Fasted plasma insulin levels were slightly higher in the CD40^fl/fl^CD11c^cre^ mice on the NASH diet compared to WT mice (0.23 ug/mL vs 0.31 ug/mL, unpaired t-test, p = 0.0667, Supplemental Fig. 3B). Glucose and insulin sensitivity were measured after 12 weeks of diet and were not affected in the CD40^fl/fl^CD11c^cre^ mice and WT mice on the NASH diet as shown by no differences between the groups during the GTT and ITT (Supplemental Fig. 3C,D). Plasma cholesterol and TG did not significantly differ between the groups on the same diet (Supplemental Fig. 3E,F).

### CD40^fl/fl^CD11c^cre^ mice have reduced liver inflammation during NASH compared to WT mice

**Figure 6:**
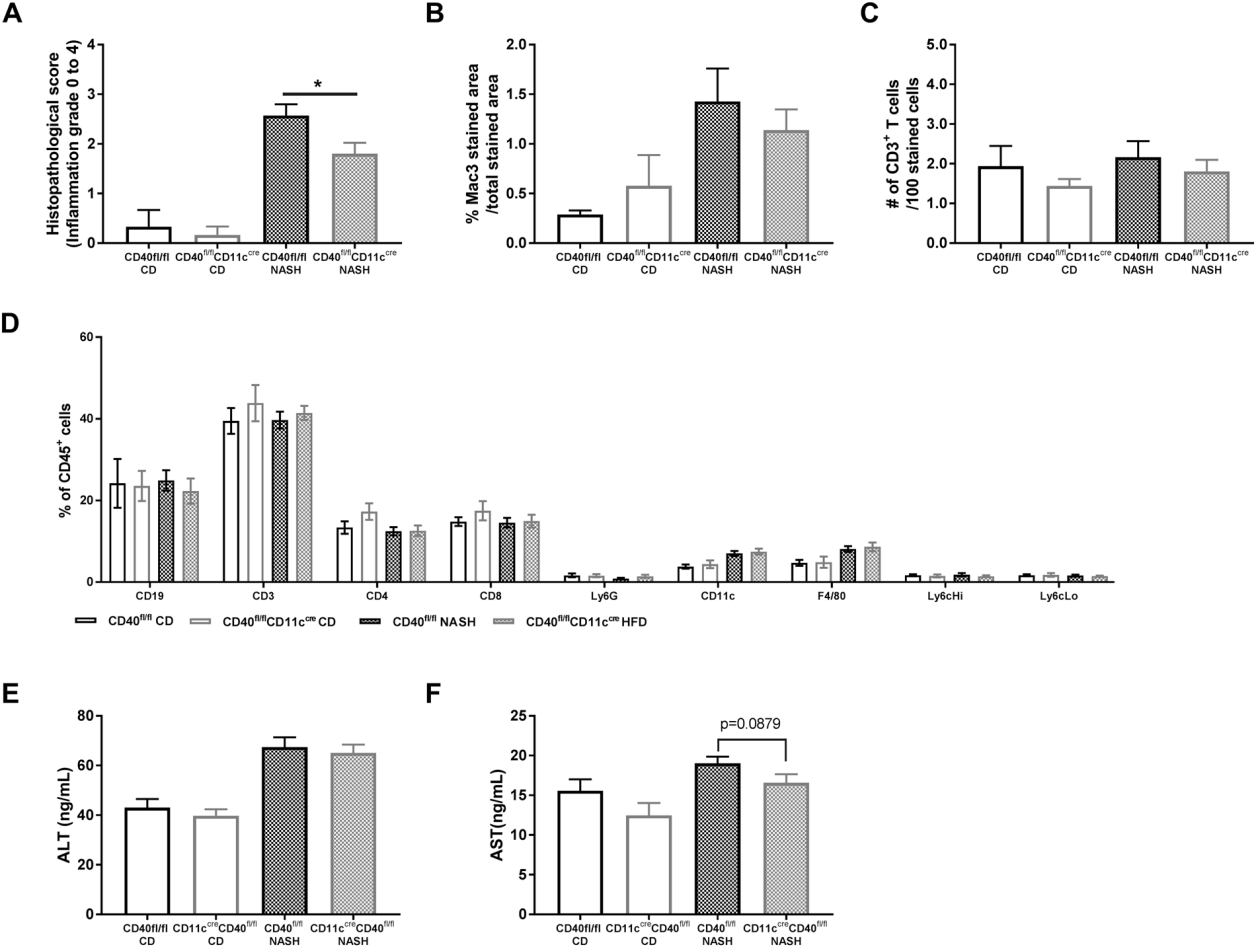
Liver inflammation is lower in CD40^fl/fl^CD11c^cre^ compared to WT mice on a NASH inducing diet. (**A**) Mean histopathological inflammation grade. (**B**) quantification of Mac3 staining in the liver (**C**) quantification of CD3 staining in the liver. (**D**) Liver leukocyte subsets analyzed by FACS. (**E**) plasma ALT levels. (**F**) plasma AST levels. Data is represented as mean ± SEM. *P < 0.05 for comparison between WT and CD40^fl/fl^CD11c^cre^ mice fed a similar diet. n = 6/group for CD, and n = 15/group for NASH diet.

Although no differences could be observed in hepatosteastosis after the NASH diet, the degree of hepatic inflammation had decreased in CD40^fl/fl^CD11c^cre^ mice compared to WT mice (Mann-Whitney test, p = 0.0301, Fig. 6A). Quantification of liver Mac-3 staining and CD3 staining shows that the number of macrophages and T cells in the liver of CD40^fl/fl^CD11c^cre^ mice are not significant different between the groups, but are both slightly decreased compared to WT mice on a NASH diet, accounting for the total decrease in inflammation (Fig. 6B,C). FACS data of the liver shows no difference in leukocyte composition (Fig. 6D). ALT levels had increased after feeding the NASH inducing diet but did not differ between WT and CD40^fl/fl^CD11c^cre^ mice (Fig. 6E), whereas AST levels were lower in CD40^fl/fl^CD11c^cre^ mice (19.02 ng/mL vs 16.57 ng/mL, unpaired t-test, p = 0.0879, Fig. 6F). Furthermore, systemic inflammation was not different between the groups on the same diet (blood, spleen and lymph node leukocyte subsets, Supplemental Fig. 4A–C).

## Discussion

In line with the worsened metabolic phenotype observed in CD40^−/−^ mice on a high fat diet^10–13^, we here show that HFD fed CD40^fl/fl^CD11c^cre^ mice become more obese than WT mice, develop hyperinsulinemia and have an increased lipid uptake in adipose tissue and liver, resulting in severe hepatic steatosis. In contrast to the phenotype observed in total body CD40-deficient mice, CD40-deficiency in CD11c^+^ cells did not result in a profound increase in pro-inflammatory mediators in lymphoid organs, adipose tissue or liver. However, upon high fat diet feeding CD40^fl/fl^CD11c^cre^ mice did show a reduction in the number of CD4^+^FoxP3^+^ regulatory T cells (Tregs) in blood, adipose tissue, liver and lymphoid organs.

Tregs function as a control mechanism that can affect the behavior of other T cell populations and suppress overactive immune responses^26^. CD40-deficient mice are known to have a reduced amount of Tregs in peripheral blood, spleen and the thymus^27,28^. Especially B cell CD40 is involved in survival of Tregs in the thymus^29^, whereas deficiency of CD11c-CD40 resulted in only a minor decrease in natural Tregs in the thymus^30^. In our experiment, we could not observe decreases in Treg levels in blood, lymphoid organs and tissues in CD40^fl/fl^CD11c^cre^ upon SFD, but we did see a HFD induced reduction of Tregs in lymphoid organs, and especially in the liver. This suggests that CD40^+^CD11c^+^ cells are particularly involved in the reactive generation of Tregs during chronic inflammatory conditions^31^, and can be generated through homeostatic proliferation of CD4^+^CD25^−^ T cells^32^.

Although we did not observe signs of enhanced inflammation, the decrease in Tregs was surprisingly accompanied by an increase in systemic and hepatic cholesterol levels in CD40^fl/fl^CD11c^cre^ mice fed a HFD. Consistent with our findings, depleting FoxP3^+^ Tregs in a mouse model for atherosclerosis resulted in increased plasma cholesterol levels but did not induce inflammation in the liver or the atherosclerotic plaques^33^. In this study Treg depletion reduced protein expression of the VLDL binding protein sortilin-1 in the liver increasing lipoprotein catabolism activity in the plasma, which results in accumulation of VLDL and CM lipoproteins in the circulation^33^. In our experiment we observed upregulated expression of the LDLr, SREBP2 and FAS gene suggesting that the liver takes up more cholesterol from the blood and has increased cholesterol biosynthesis^34^, while reduced gene expression of LXRα (and Idol) and its target genes indicate decreased excretion of cholesterol from hepatocytes^35^. Together this could result in the accumulation of cholesterol in the liver. Another contributor to the increased hepatosteatosis in our DIO model could be the increased leptin levels in the CD40^fl/fl^CD11c^cre^ mice. The adipose tissue hormone leptin negatively affects Treg proliferation^36,37^. Increased leptin levels contribute to the progression from NAFLD to NASH via activation of macrophage and Kupffer cells and increased oxidative stress^38^.

Interestingly, mice deficient in the co-stimulatory molecule CD80/86 (B7.1/B7.7) showed increased liver steatosis when fed a HFD, a similar phenotype as observed in our CD40^fl/fl^CD11c^cre^ mice^39^. CD80/CD86 is required for proper Treg development and the phenotype observed in these mice was also attributed to the reduction of Treg numbers in B7.1/B7.7 double knock out mice^39^. Together with our results these findings underline the important role for Tregs in obesity associated liver disease and indicate that manipulation of co-stimulatory molecule signaling to increase Treg numbers is therapeutically interesting for the prevention of hepatosteatosis and eventually NASH.

Tregs have been described to play an important role in hepatic steatosis related diseases, particularly in NAFLD and NASH. NAFLD and NASH are important health problems related to obesity and NAFLD affects 25% of the global adult population, of which 59% of the patients exhibits NASH^18^. NAFLD is defined by the presence of ≥5% of hepatic steatosis, and when hepatocyte damage develops, disease progresses into NASH, characterized by hepatic fibrosis^18^. Hepatic steatosis and NASH are associated with reduction of hepatic Treg numbers in both human and mouse^40,41^. Depletion of Tregs is caused by increased oxidative stress in fatty livers inducing Treg apoptosis and impairs suppression of inflammatory responses^40^. Activation of inflammatory signaling through the TNF‐α pathway contributes to the progression from simple steatosis to steatohepatitis^40^. Interestingly, pharmacological upregulation of Treg numbers, using 3, 3′-diindolylmethane (DIM), can reduce hepatic inflammation^42^.

However, when NASH was induced in our CD40^fl/fl^CD11c^cre^ mouse model, we could not observe a reduction in Tregs in liver or lymphoid organs. Moreover, absence of CD11c^+^CD40^+^ cells did not affect the degree of hepatosteatosis, which was massive (~40%) in the NASH model. These data indicate that the reduced Treg numbers observed in the DIO study were probably caused by the adipose tissue driven ectopic lipid accumulation, rather than the intrinsic effects of the liver fat during NASH. Interestingly, absence of CD11c^+^CD40^+^ cells did reduce hepatic inflammation during NASH, indicating that CD11c^+^CD40^+^ cells have a strong (lipid driven) immune-modulatory function.

In conclusion, we here show that CD40^+^CD11c^+^ cells play a crucial role in protection against obesity-induced liver steatosis via activation of regulatory T cells, thereby inducing a tolerogenic immune response that prevents hepatosteatosis.

## Material and Methods

### Mice

To study DC and CD11c+ macrophage-CD40 depletion in DIO and NASH CD40^fl/fl^CD11c^cre^ mice were generated by crossbreeding CD11c-cre transgenic mice (B6.Cg-Tg(Itgax-cre)1-1Reiz/J, purchased from Jackson Laboratory) with CD40^flfl^ mice (CD40^fl/fl^CD11c^cre^ mice). CD40^flfl^ mice were generated by custom-design at Ozgene Pty Ltd (Bentley, Australia)^43^. CD40^flfl^ mice carry loxP-sites before CD40 exon2 and after CD40 exon3. Cre-mediated recombination by the CD11c-cre mice removes the loxed CD40 exons, resulting in a non-functional CD40 peptide on CD11c^+^ cells. The CD11c-cre recombinase is highly present on dendritic cells (~80–100%) and to a smaller extent on mature macrophages (~40–75%), monocytes (~20–40%), and on lymphocytes (B and T-cells < 20%)^44^. CD40^flfl^CD11c^WT^ (WT) littermates were used as the control group. All mice were bred and maintained at the animal facility of the Academic Medical Centre, Amsterdam (AMC).

### Study design

CD40^fl/fl^CD11c^cre^ and WT mice (male, age 6–8 weeks) were fed a high fat diet (HFD; SNIFF-D12492 Energy 22% from carbohydrates, 24% from protein, and 54% from fat), or a standard fat diet (SFD; SNIFF-D12450B Energy 65% from carbohydrates, 26% from protein, and 9% from fat) for 18 weeks to study obesity and insulin resistance. Another group of CD40^fl/fl^CD11c^cre^ and WT mice (male, age 6–8 weeks) were fed a NASH inducing diet (NASH, D17010103 Research diets, Energy 40% from fat (mostly partially hydrogenated corn oil), 20% from fructose, and 2% from cholesterol)) or control diet (CD, D17072805, Research diets, Energy 10% from fat, 70% from carbohydrates (no fructose), and 20% from protein) for 20 weeks to study nonalcoholic steatohepatitis.

Mice had *ad libitum* access to food and water and were maintained under a 12-hour light-dark cycle. Body weight was monitored weekly. At the end of the dietary exposure period mice were euthanized, and blood samples were collected after intracardiac puncture using EDTA coated syringes. Liver, epidydimal adipose tissue (EpAT), muscle, spleen, and lymph nodes were removed after sacrifice and used for subsequent analyses. All the experimental procedures were approved by the Ethical Committee for Animal Experiments of the Academic Medical Centre, Amsterdam (AMC). All experiments were performed in accordance with relevant guidelines and regulations.

### Glucose and insulin tolerance test

After 13 weeks of dietary exposure a glucose (GTT) and insulin tolerance test (ITT) were performed. For the GTT, mice were fasted for 6 hours and injected i.p. with glucose (1 mg/g body weight, Sigma-Aldrich, Saint Louis, MO, USA). For the ITT 4 hour fasted mice were injected i.p. with insulin (1,1mU/g body weight, Sigma-Aldrich). Blood glucose levels were measured from whole blood using a glucometer (Bayercontour, Basel, Switzerland) at times indicated in the figures.

### Plasma lipids, insulin, adipokine and liver enzyme measurements

Fasting plasma insulin levels were measured in samples from 6 hour fasted mice by using an insulin ELISA kit (Mercodia, Uppsala, Sweden) following manufacturers’ protocol. Plasma leptin and adiponectin levels of the DIO mice were measured using a mouse leptin ELISA Kit (ChrystalChem) and a mouse adiponectin ELISA Kit (AssayPro). Liver enzymes were measured in plasma samples using an alanine aminotransferase (ALT) activity kit (DIO mice, Sigma-Aldrich) or ELISA Kit (NASH mice, Biomatik), a mouse aspartate aminotransferase (AST) ELISA Kit (Biomatik), a BCG (Bromocresol Green) albumin assay kit (Sigma-Aldrich), a glutamate dehydrogenase (GDH) activity assay kit (Sigma-Aldrich), and a bilirubin assay kit (Sigma-Aldrich).

### Cholesterol and triglyceride measurements

Total cholesterol and triglyceride (TG) concentrations in plasma and liver were measured by standard enzymatic methods (CHOD-PAP and GPO-PAP; Roche Diagnostics). Frozen liver tissues were homogenized in 1 ml of SET buffer (250 mM sucrose, 2 mM EDTA, and 10 mM Tris), and the samples then underwent multiple freeze-thaw cycles for cell-destruction. Individual plasma lipoprotein levels were measured by fast-performance liquid chromatography as described before^45^.

### Flow cytometry

White blood cells from blood, spleen, lymph nodes (LN), liver and stromal vascular fraction (SVF) from EpAT were analyzed by flow cytometry. EpAT was minced into small pieces and digested with liberase (0.25 mg/mL, Roche) for 45 minutes at 37 °C. The digested samples were passed through a 70-µm nylon mesh (BD Biosciences, Breda, the Netherlands). The SVF was obtained from the resulting pellet and resuspended in FACS buffer. Erythrocytes in blood and spleen were removed by incubation with hypotonic lysis buffer (8.4 g of NH4Cl and 0.84 g of NaHCO3 per liter of distilled water). To prevent non-specific binding of antibodies to the Fc receptor, all cell suspensions were incubated with CD16/32 antibody (eBioscience, San Diego, CA, USA) in FACS buffer (0.5% BSA, 0.01% NaN_3_ in PBS) before the antibodies were incubated with the indicated tissues. Fluorescence was measured by flow cytometry (FACSCanto II, BD Biosciences, Breda, The Netherlands) and analyzed with FlowJo software version 7.6.5. (Tree star). The antibodies used are listed in Supplemental Table 1.

### Histology

EpAT and liver tissues were collected, fixed in 4% paraformaldehyde and embedded in paraffin. Liver steatosis was graded on 4 μm thick haematoxylin-eosin (H&E) stained sections. Grades were scored in a range from 0–5 were 0 = no steatosis, 1 = minimal steatosis, 2 = mild steatosis, 3 = moderate steatosis, 4 = severe steatosis, and 5 = marked steatosis. Immunohistochemistry (IHC) on liver and EpAT was performed for antibodies indicated in the graphs and listed in Supplemental Table 1. IHC staining was quantified using Image J. Adipocyte size from DIO mice was measured on EpAT H&E stained sections using image J (NIH, Bethesda, Maryland, USA). Additionally, livers from DIO mice were frozen, embedded in OCT and frozen at −80 degrees Celsius. To measure liver lipid content, 5 μm thick cryosections of the liver were stained with Oil red O (Sigma-Aldrich, Zwijndrecht, the Netherlands). Analyses were performed by an observer who was blinded for the experimental conditions.

### Gene expression analysis

Total RNA of EpAT and liver was extracted using TRIzol (Invitrogen, Carlsbad, CA, USA), while total RNA from muscle was extracted using the GeneJET RNA Purification Kit (Thermo Scientific, Massachusetts, USA). RNA was reverse transcribed with an iScript cDNA synthesis kit (Bio-Rad, Veenendaal, the Netherlands). qPCR was performed using a SYBR green PCR kit (Applied Biosystems, Leusden, the Netherlands) on a ViiA7 real-time PCR system (Applied Biosystems). Primer sequences can be found in Supplemental Table 2.

### Statistical analysis

The experiments were performed with n = 8 mice (HFD), n = 7 mice (SFD), n = 15 mice (NASH), and n = 6 mice (CD), and results are presented as means ± SEM. Data were analyzed by an unpaired *t*-test, a Mann-Whitney test when appropriate, or a two-way ANOVA when comparing multiple groups using GraphPad Prism 7.0 software (GraphPad Software, Inc., La Jolla, CA, USA). P-values < 0.05 were considered significant.

## Supplementary information

Supplemental figures

## Data Availability

All data generated or analyzed during this study are included in this published article (and its Supplementary Information Files).

## References

[1] WHO. *Obesity and overweight*, http://www.who.int/mediacentre/factsheets/fs311/en/ (2018).

[2] Huang W (2010). Depletion of liver Kupffer cells prevents the development of diet-induced hepatic steatosis and insulin resistance. Diabetes.

[3] Weisberg SP (2003). Obesity is associated with macrophage accumulation in adipose tissue. J. Clin. Invest..

[4] Kang YE (2016). The Roles of Adipokines, Proinflammatory Cytokines, and Adipose Tissue Macrophages in Obesity-Associated Insulin Resistance in Modest Obesity and Early Metabolic Dysfunction. PLoS One.

[5] Knights AJ, Funnell APW, Pearson RCM, Crossley M, Bell-Anderson KS (2014). Adipokines and insulin action: A sensitive issue. Adipocyte.

[6] Grewal IS, Flavell RA (1996). The role of CD40 ligand in costimulation and T-cell activation. Immunol Rev..

[7] Pan P-Y (2010). Immune Stimulatory Receptor CD40 Is Required for T-Cell Suppression and T Regulatory Cell Activation Mediated by Myeloid-Derived Suppressor Cells in Cancer. Cancer Res..

[8] Poggi M (2011). CD40L deficiency ameliorates adipose tissue inflammation and metabolic manifestations of obesity in mice. Arterioscler Thromb Vasc Biol.

[9] Wolf D (2012). CD40L deficiency attenuates diet-induced adipose tissue inflammation by impairing immune cell accumulation and production of pathogenic IgG-antibodies. PLoS One.

[10] Chatzigeorgiou A (2014). Blocking CD40-TRAF6 signaling is a therapeutic target in obesity-associated insulin resistance. Proc. Natl. Acad Sci. USA.

[11] Guo CA (2013). CD40 deficiency in mice exacerbates obesity-induced adipose tissue inflammation, hepatic steatosis, and insulin resistance. Am J. Physiol. Endocrinol. Metab..

[12] Wolf D (2014). Coinhibitory suppression of T cell activation by CD40 protects against obesity and adipose tissue inflammation in mice. Circulation.

[13] Yi Z, Stunz LL, Bishop GA (2014). CD40-mediated maintenance of immune homeostasis in the adipose tissue microenvironment. Diabetes.

[14] Schonbeck U, Libby P (2001). The CD40/CD154 receptor/ligand dyad. Cell Mol Life Sci..

[15] Poggi M (2009). The inflammatory receptor CD40 is expressed on human adipocytes: contribution to crosstalk between lymphocytes and adipocytes. Diabetologia.

[16] Poltorak MP, Schraml BU (2015). Fate mapping of dendritic cells. Front Immunol..

[17] Stefanovic-Racic M (2012). Dendritic cells promote macrophage infiltration and comprise a substantial proportion of obesity-associated increases in CD11c+ cells in adipose tissue and liver. Diabetes.

[18] Younossi ZM (2016). Global epidemiology of nonalcoholic fatty liver disease—Meta‐analytic assessment of prevalence, incidence, and outcomes. Hepatology.

[19] Wentworth JM (2010). Pro-inflammatory CD11c+CD206+ adipose tissue macrophages are associated with insulin resistance in human obesity. Diabetes.

[20] Morris DL (2016). CD40 promotes MHC class II expression on adipose tissue macrophages and regulates adipose tissue CD4+ T cells with obesity. J. Leukoc. Biol..

[21] de Ferranti S, Mozaffarian D (2008). The perfect storm: obesity, adipocyte dysfunction, and metabolic consequences. Clin Chem..

[22] Parlee SD, Lentz SI, Mori H, MacDougald OA (2014). Quantifying size and number of adipocytes in adipose tissue. Methods Enzymol.

[23] Ioannou GN (2016). The Role of Cholesterol in the Pathogenesis of NASH. Trends Endocrinol Metab.

[24] Ozer J, Ratner M, Shaw M, Bailey W, Schomaker S (2008). The current state of serum biomarkers of hepatotoxicity. Toxicology.

[25] O’Brien PJ, Slaughter MR, Polley SR, Kramer K (2002). Advantages of glutamate dehydrogenase as a blood biomarker of acute hepatic injury in rats. Lab Anim.

[26] Feuerer M (2009). Lean, but not obese, fat is enriched for a unique population of regulatory T cells that affect metabolic parameters. Nat. Med..

[27] Guiducci C, Valzasina B, Dislich H, Colombo MP (2005). CD40/CD40L interaction regulates CD4+CD25+ T reg homeostasis through dendritic cell-produced IL-2. Eur J Immunol.

[28] Martin S, Agarwal R, Murugaiyan G, Saha B (2010). CD40 expression levels modulate regulatory T cells in Leishmania donovani infection. J. Immunol..

[29] Yamano T (2015). Thymic B Cells Are Licensed to Present Self Antigens for Central T Cell Tolerance Induction. Immunity.

[30] Garg G (2017). Unique properties of thymic antigen-presenting cells promote epigenetic imprinting of alloantigen-specific regulatory T cells. Oncotarget.

[31] Povoleri GA (2013). Thymic versus induced regulatory T cells - who regulates the regulators?. Front Immunol.

[32] Curotto de Lafaille MA, Lino AC, Kutchukhidze N, Lafaille JJ (2004). CD25− T cells generate CD25+Foxp3+ regulatory T cells by peripheral expansion. The Journal of Immunology.

[33] Klingenberg R (2013). Depletion of FOXP3+ regulatory T cells promotes hypercholesterolemia and atherosclerosis. J. Clin Invest..

[34] Goldstein JL, Brown MS (2009). The LDL receptor. Arterioscler Thromb Vasc Biol.

[35] Baranowski M (2008). Biological role of liver X receptors. J. Physiol Pharmacol.

[36] De Rosa V (2007). A key role of leptin in the control of regulatory T cell proliferation. Immunity.

[37] Matarese G, Procaccini C, De Rosa V, Horvath TL, La Cava A (2010). Regulatory T cells in obesity: the leptin connection. Trends Mol Med.

[38] Chatterjee S (2013). Leptin is key to peroxynitrite-mediated oxidative stress and Kupffer cell activation in experimental nonalcoholic steatohepatitis. J Hepatol.

[39] Chatzigeorgiou A (2014). Dual role of B7 costimulation in obesity‐related nonalcoholic steatohepatitis and metabolic dysregulation. Hepatology.

[40] Ma X (2007). A high‐fat diet and regulatory T cells influence susceptibility to endotoxin‐induced liver injury. Hepatology.

[41] Rau M (2016). Progression from nonalcoholic fatty liver to nonalcoholic steatohepatitis is marked by a higher frequency of Th17 cells in the liver and an increased Th17/resting regulatory T cell ratio in peripheral blood and in the liver. The Journal of Immunology.

[42] Liu Y (2014). 3, 3′-diindolylmethane alleviates steatosis and the progression of NASH partly through shifting the imbalance of Treg/Th17 cells to Treg dominance. International Immunopharmacology.

[43] Aarts SABM (2018). Macrophage CD40 plays a minor role in obesity-induced metabolic dysfunction. PLoS One.

[44] Abram CL, Roberge GL, Hu Y, Lowell CA (2014). Comparative analysis of the efficiency and specificity of myeloid-Cre deleting strains using ROSA-EYFP reporter mice. J Immunol Methods.

[45] Seijkens Tom, Hoeksema Marten A., Beckers Linda, Smeets Esther, Meiler Svenja, Levels Johannes, Tjwa Marc, de Winther Menno P. J., Lutgens Esther (2014). Hypercholesterolemia-induced priming of hematopoietic stem and progenitor cells aggravates atherosclerosis. The FASEB Journal.

