# Supplementary material for: Depletion of CD40 on CD11c+ cells worsens the metabolic syndrome and ameliorates hepatic inflammation during NASH
Source: Sci Rep. 2019 Oct 11;9:14702. doi: 10.1038/s41598-019-50976-6 (PMC6789104; doi:10.1038/s41598-019-50976-6)
Supplement: Supplementary file 1 — Supplemental figures [file 41598_2019_50976_MOESM1_ESM.pdf]

Depletion of CD40 on CD11c$^+$ cells worsens the metabolic syndrome and ameliorates hepatic inflammation during NASH.

Suzanne Aarts[1], Myrthe Reiche[1], Myrthe den Toom[1], Marion Gijbels[1,2], Linda Beckers[1], Norbert Gerdes[3], Esther Lutgens[1,4*]

1. Department of Medical Biochemistry, Subdivision of Experimental Vascular Biology, Amsterdam University Medical Centers, location AMC, Amsterdam Cardiovascular Sciences (ACS), University of Amsterdam, Amsterdam, The Netherlands
2. Department of Pathology and Department of Molecular Genetics, CARIM, University of Maastricht, Maastricht, The Netherlands
3. Division of Cardiology, Pulmonology, and Vascular Medicine, Medical Faculty, University Hospital Düsseldorf, Düsseldorf, Germany
4. Institute for Cardiovascular Prevention (IPEK), Ludwig Maximilian University (LMU), Munich, Germany

***Corresponding author**: Prof. Esther Lutgens, Department of Medical Biochemistry, subdivision Experimental Vascular Biology, Academic Medical Centre, University of Amsterdam, Meibergdreef 15, 1105 AZ, Amsterdam, The Netherlands. Tel. +31 (0)20 5 66 33 80. E-mail: e.lutgens@amc.uva.nl and Institute for Cardiovascular Prevention (IPEK), Ludwig Maximilian University (LMU), Pettenkoferstraße 9, 80336 Munich, Germany. Tel. +49-(0)89-44005-4350. E-mail: Esther.Lutgens@med.uni-muenchen.de.

Supplemental Fig. 1. Adipose tissue inflammation is not affected in CD40$^{fl/fl}$CD11c^{cre} mice with DIO. (A) Total number of CD45^{+} cells measured in EpAT by flow cytometry. (B) Quantification of histological staining of macrophages (CD68^{+}) and T cells (CD3^{+}) in EpAT. (C) Total number of myeloid leukocyte subsets measured in EpAT. (D) Total number of lymphoid leukocyte subsets measured in EpAT. Data is represented as mean ± SEM for comparison between WT and CD40$^{fl/fl}$CD11c^{cre} mice fed the same diet. *n= 7/group for SFD, and n=8/group for HFD.*

Supplemental Fig. 2. EpAT mRNA expression DIO mice. (A) pro-inflammatory genes. (B) anti-inflammatory genes. Data are represented as mean ± SEM. **P<0.01. *n=8/group for HFD.*

Supplemental Fig. 3. CD40$^{fl/fl}$CD11c^{cre} mice have slightly worsened insulin resistance compared to WT mice on the NASH diet. (A) Fasted glucose levels (B) Fasted insulin levels (C) glucose levels during glucose tolerance test (GTT), (D) glucose levels during insulin tolerance test (ITT). (E) Plasma total cholesterol. (F) Plasma triglycerides. *n= 3/group for CD, and n=11/group for NASH diet.*

Supplemental Fig. 4. Systemic inflammation after control and NASH diet. (A) Blood. (B) Spleen. (C) Lymph nodes.

Supplemental Table 1. List of antibodies

Supplemental Table 2. Primer sequences

Supplemental Fig. 1. Adipose tissue inflammation is not affected in CD40$^{fl/fl}$CD11c^{cre} mice with DIO.

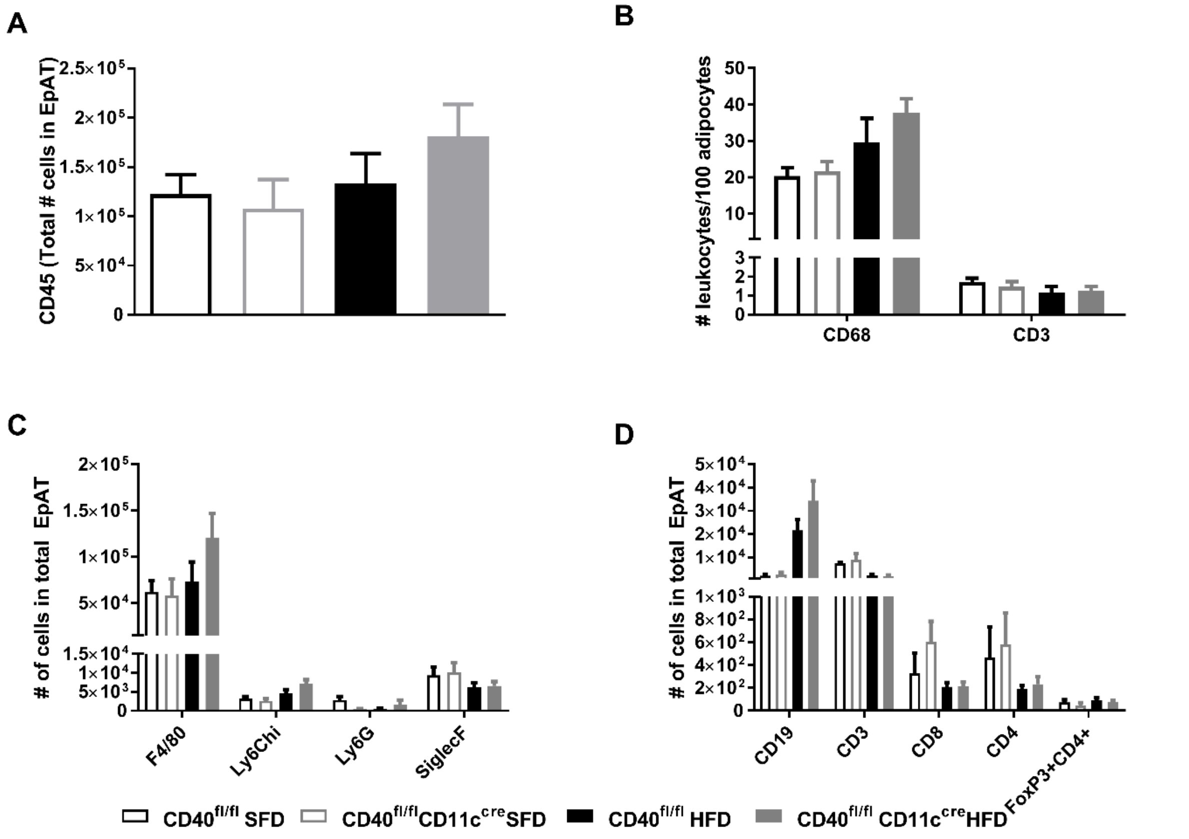

Supplemental Fig. 2. EpAT mRNA expression DIO mice.

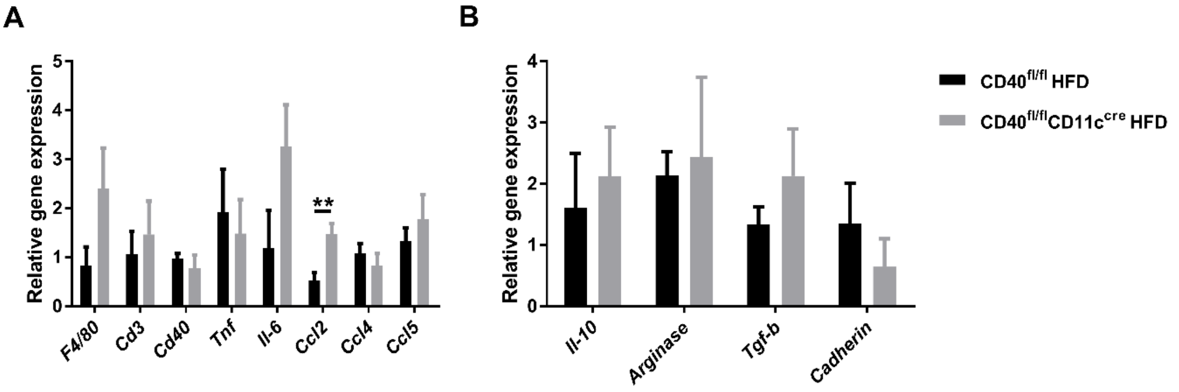

Supplemental Fig. 3. CD40$^{fl/fl}$CD11c^{cre} mice have slightly worsened insulin resistance compared to WT mice on the NASH diet.

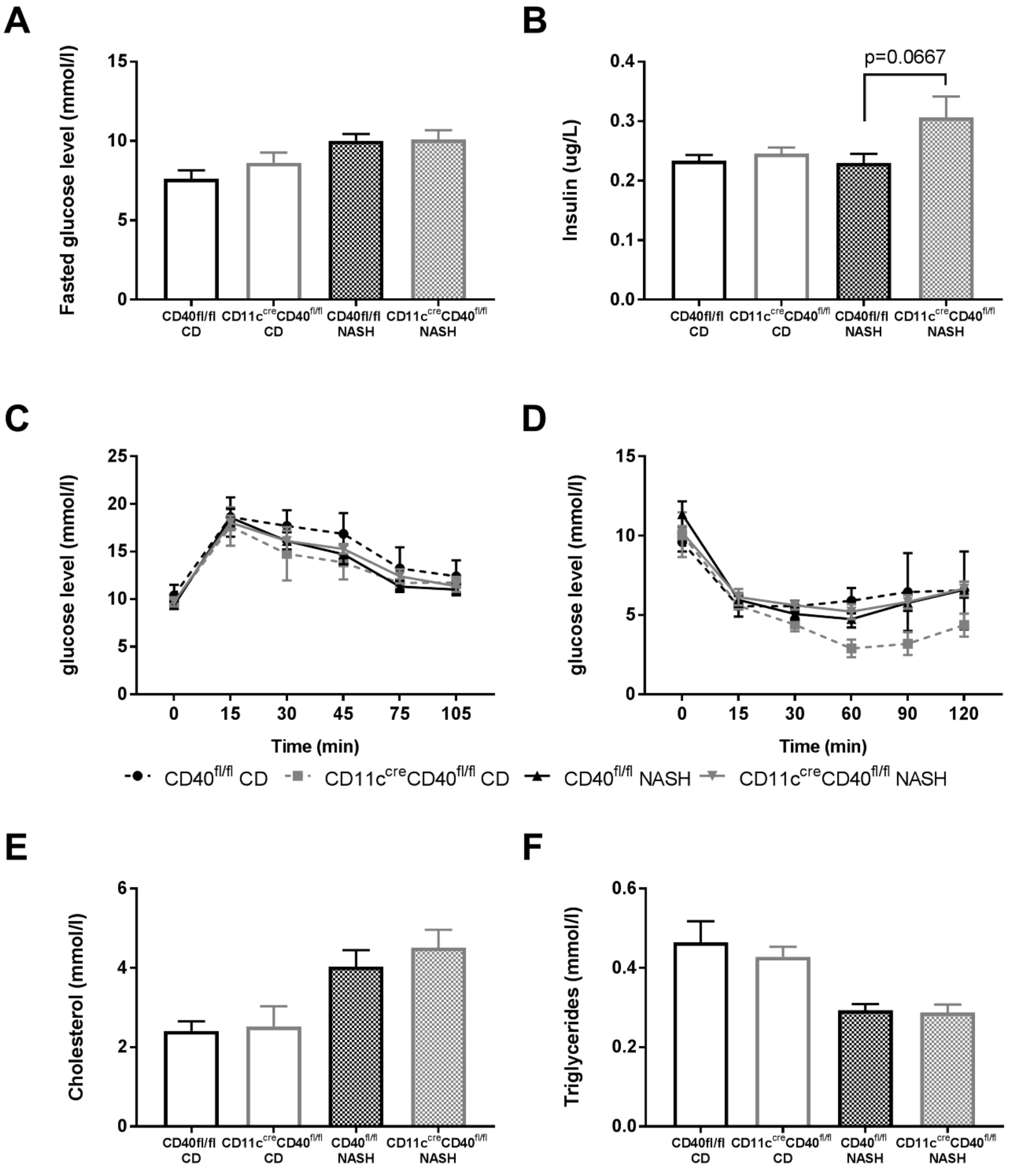

Supplemental Fig. 4. Systemic inflammation after control and NASH diet.

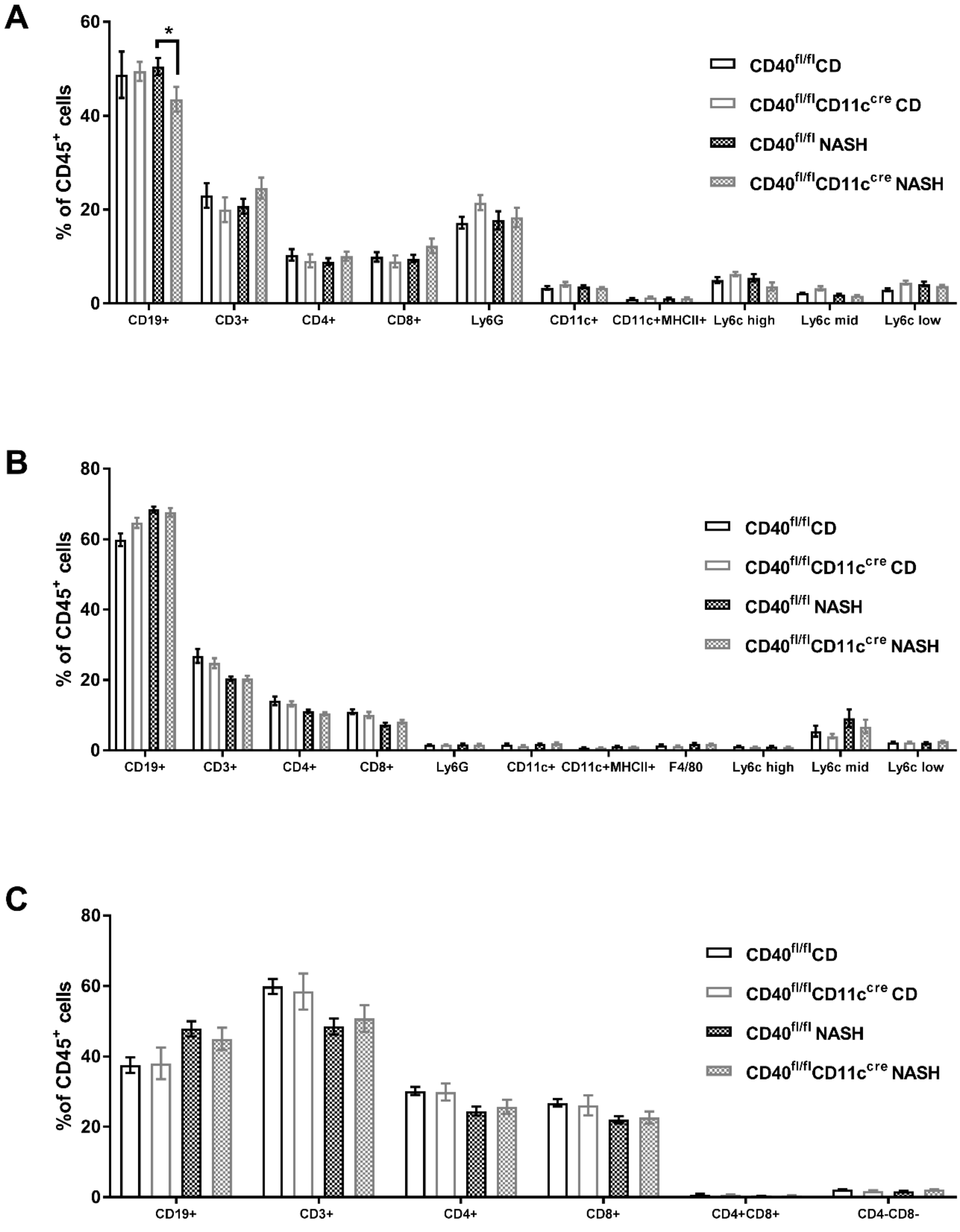

Supplemental Tables

Table 1. List of antibodies

CD16/32	eBioscience	San Diego, CA, USA
CD45	eBioscience	San Diego, CA, USA
CD19	eBioscience	San Diego, CA, USA
CD3	BioLegend	San Diego, CA, USA
CD4	BD	Breda, the Netherlands
CD8	eBioscience	San Diego, CA, USA
FoxP3	eBioscience	San Diego, CA, USA
CD11b	BD	Breda, the Netherlands
Ly6G	BD	Breda, the Netherlands
F4/80	AbD serotec	Puchheim, Germany
Ly6C	eBioscience	San Diego, CA, USA
CD45 (IHC)	BD	Breda, the Netherlands
Mac-3 (IHC)	BD	Breda, the Netherlands
CD68 (IHC)	AbD serotec	Puchheim, Germany
CD3 (IHC)	AbD serotec	Puchheim, Germany

Table 2. Primer sequences

	**Forward**	**Reverse**
**RorγT**	5'-ACAGCCACTGCATTCCCAGTTT-3'	5'-TCTCGGAAGGACTTGCAGACAT-3'
**Il-17**	5'-TCCCTCTGTGATCTGGGAAG-3'	5'-CTCGACCCTGAAAGTGAAGG-3'
**Lxr-α**	5'-GCTCTGCTCATTGCCATCAG-3'	5'-TGTTGCAGCCTCTCTACTTGGA-3'
**Idol**	5'-AGGAGATCAACTCCACCTTCTG-3'	5'-ATCTGCAGACCGGACAGG-3'
**Abca1**	5'-CCCAGAGCAAAAAGCGACTC-3'	5'-GGTCATCATCACTTTGGTCCTTG-3'
**Abcg1**	5'-TCTGATGGCCGCTTTCTCGG-3'	5'-GACACGACCTCGTCCACTGA-3'
**Abcg8**	5'-AACCCTGCGGACTTCTACG-3'	5'-CTGCAAGAGACTGTGCCTTCT-3'
**Srebp1c**	5'-CCGGCTATTCCGTGAACATC-3'	5'-ATCCAAGGGCATCTGAGAACTC-3'
**Fas**	5'-TGCTCCCAGCTGCAGGC-3'	5'-GCCCGGTAGCTCTGGGTGTA-3'
**Acc**	5'-GGAGATGTACGCTGACCGAG-3'	5'-TACCCGACGCATGGTTTTCA-3'
**Elovl6**	5'-CCCGAACTAGGTGACACGAT-3'	5'-CCAGCGACCATGTCTTTGTA-3'
**Scd1**	5'-CCTTCCCCTTCGACTACTCTG-3'	5'-GCCATGCAGTCGATGAAGAA-3'
**Srepf2**	5'-TAACCCCTTGACTTCCTTGC-3'	5'-CACACCATTTACCAGCCACA-3'
**Hmgcs1**	5'-TGATCCCCTTTGGTGGCTGA-3'	5'-AGGGCAACGATTCCCACATC-3'
**Hmgcr**	5'-TCTGGCAGTCAGTGGGAACTATT-3'	5'-CCTCGTCCTTCGATCCAATTT-3'
**Ldl-r**	5'-GCATCAGCTTGGACAAGGTGT-3'	5'-GGGAACAGCCACCATTGTTG-3'
**Sqle**	5'-GACCTCGTTCGTGACGGAC-3'	5'-CTCCCCAACTATCCTGTCGG-3'

**Glut2**	5'-TCATTCTTTGGTGGGTGGCT-3'	5'-CCAGTCCTGAAATTAGCCCACAA-3'
**Chrebp**	5'-CCTCACTTCACTGTGCCTCA-3'	5'-ACAGGGGTTGTTGTCTCTGG-3'
**Dgat2**	5'-TGGTGTGTGGTGATGCTGATC-3'	5'-GCCAGGCGCTTCTCAA-3'
**Cyp7a1**	5'-CAGGGAGATGCTCTGTGTTCA-3'	5'-AGGCATACATCCCTTCCGTGA-3'
**CD36**	5'-GCAAAGAACAGCAGCAAAATC-3'	5'-CAGTGAAGGCTCAAAGATGG-3'
**Sort1**	5'-TGTGGCCAAGCAGCCATCCG-3'	5'-TCAGGCTGCTCCACGCACTC-3'
**F4/80**	5'-CTTTGGCTATGGGCTTCCAGTC-3'	5'-GCAAGGAGGACAGAGTTTATCGTG-3'
**CD3**	5'-AACACGTACTTGTACCTGAAAGCTC-3'	5'-GATGATTATGGCTACTGCTGTCA-3'
**CD40**	5'-GAGTCAGACTAATGTCATCTGTGGTT-3'	5'-ACCCCGAAAATGGTGATG-3'
**Tnf**	5'-CATCTTCTCAAAATTCGAGTGACAA-3'	5'-TGGGAGTAGACAAGGTACAACCC-3'
**IL-1b**	5'AAAGAATCTATACCTGTCCTGTGTAATGAAA-3'	5'-GGTATTGCTTGGGATCCACACT-3'
**Il-6**	5'-GCTACCAAACTGGATATAATCAGGAAA-3'	5'CTTGTTATCTTTTAAGTTGTTCTTCATGTACTC-3'
**IL-12b**	5'-GGTGCAAAGAAACATGGACTTG-3'	5'-CACATGTCACTGCCCGAGAGT-3'
**Ccl2**	5' AGCACCAGCCAACTCTCACT 3'	5'-CGTTAACTGCATCTGGCTGA-3'
**Ccl4**	5'-GAAGCTTTGTGATGGATTACTATGAGA-3'	5'-GTCTGCCTCTTTTGGTCAGGAA-3'
**Ccl5**	5'-GGAGTATTTCTACACCAGCAGCAA-3'	5'-GCGGTTCCTTCGAGTGACA-3'
**Nos2**	5'-GCAAACCCAAGGTCTACGTTCA-3'	5'-CCTCATTGGCCAGCTGCTT-3'
**MHCII**	5'-AGCCCCATCACTGTGGAGT-3'	5'-GATGCCGCTCAACATCTTGC-3'
**Il-10**	5'-TTTGAATTCCCTGGGTGAGAA-3'	5'-CTCCACTGCCTTGCTCTTATTTTC-3'
**Arginase**	5'-CCTCCCTGCCAATCATGTTC-3'	5'-TCAGGACGCAAGGAATTTGC-3'
**Tgf-β**	5'-GCCCTTCCTGCTCCTCATG-3'	5'-CCGCACACAGCAGTTCTTCTC-3´
**Cadherin**	5'-ATAATGACGCAGCTCAAGAATCTC-3'	5'-TTGTATTCGCCAATCTCTAAGTCC-3'
